# Genome-Wide Association Study of Irritable vs. Elated Mania Suggests Genetic Differences between Clinical Subtypes of Bipolar Disorder

**DOI:** 10.1371/journal.pone.0053804

**Published:** 2013-01-10

**Authors:** Tiffany A. Greenwood, the Bipolar Genome Study (BiGS) Consortium, John R. Kelsoe

**Affiliations:** 1 Department of Psychiatry, University of San Diego, La Jolla, California, United States of America; 2 San Diego Veterans Administration Healthcare System, San Diego, California, United States of America; University of Wuerzburg, Germany

## Abstract

The use of clinical features to define subtypes of a disorder may aid in gene identification for complex diseases. In particular, clinical subtypes of mania may distinguish phenotypic subgroups of bipolar subjects that may also differ genetically. To assess this possibility, we performed a genome-wide association study using genotype data from the Bipolar Genome Study (BiGS) and subjects that were categorized as having either irritable or elated mania during their most severe episode. A bipolar case-only analysis in the GAIN bipolar sample identified several genomic regions that differed between irritable and elated subjects, the most significant of which was for 33 SNPs on chromosome 13q31 (peak p = 2×10^−7^). This broad peak is in a relative gene desert over an unknown EST and between the *SLITRK1* and *SLITRK6* genes. Evidence for association to this region came predominantly from subjects in the sample that were originally collected as part of a family-based bipolar linkage study, rather than those collected as bipolar singletons. We then genotyped an additional sample of bipolar singleton cases and controls, and the analysis of irritable vs. elated mania in this new sample did not replicate our previous findings. However, this lack of replication is likely due to the presence of significant differences in terms of clinical co-morbity that were identified between these singleton bipolar cases and those that were selected from families segregating the disorder. Despite these clinical differences, analysis of the combined sample provided continued support for 13q31 and other regions from our initial analysis. Though genome-wide significance was not achieved, our results suggest that irritable mania results from a distinct set of genes, including a region on chromosome 13q31.

## Introduction

Bipolar affective disorder affects approximately 1% of the population and is characterized by episodes of major depression interspersed with periods of mania. Epidemiological data have suggested that bipolar disorder is familial with a substantial genetic component and an estimated heritability >80% [1]–[3]. Despite the apparent role for genetics in bipolar disorder, factors such as environmental influences, genetic heterogeneity, and epistatic interactions have made the identification of causal genes difficult, and the etiology of bipolar disorder remains largely a mystery. The use of clinical subphenotypes of bipolar disorder may aid in the discovery of predisposing genes by creating more homogenous groups of patients for analysis [4].

Mania is characterized by racing thoughts, rapid speech, and increased energy and activity. Several studies have found evidence for distinct subtypes of mania, which have been shown to differ in their response to treatment [5]–[7]. The classic and most common presentation of mania is elated, or euphoric, mania, which is characterized by an elevated mood. Alternatively, bipolar patients with the irritable mania subtype present with an angry, agitated, or unpleasant mood. Whereas patients with euphoric mania respond well to lithium, those with irritable mania respond poorly to lithium and show a better response to anticonvulsants [6]. These observations of clinical subtypes of mania that correlate with pharmacological response suggest that genetically distinct subtypes of bipolar disorder may exist.

Following an examination of the heritability and stability of irritable mania, we explored this hypothesis through a genome-wide association (GWA) analysis of irritable mania in bipolar subjects and controls genotyped by the Bipolar Genome Study (BiGS) as part of the Genetic Association Information Network (GAIN). Nearly two-thirds of the GAIN bipolar subjects were derived from families collected as part of the National Institute of Mental Health (NIMH) Genetics Initiative for Bipolar Disorder, with the remaining GAIN bipolar subjects collected as singletons. In a case-only analysis, 117 subjects with irritable mania were compared to 843 subjects with elated mania to identify genetic factors that may modify the expression of mania. A secondary analysis comparing subjects with irritable mania to 1,033 controls was performed to identify genetic factors that are unique to the irritable mania subtype, and these findings were contrasted with the analysis of subjects with elated mania vs. controls. The subsequent genotyping of an additional sample of singleton bipolar subjects and controls performed by the Translational Genomics Institute (TGEN) provided an independent sample of 121 and 1,026 subjects with irritable and elated mania, respectively, for replication. The irritable vs. elated mania analyses were performed in both the replication and combined samples to further investigate utility of irritable mania as a genetically distinct subtype of bipolar disorder.

## Methods

### Ethics Statement

Each collection site in the BiGS Consortium received approval for subject ascertainment, assessment, and collection of DNA for genetic studies as part of the NIMH Bipolar Disorder Genetics Initiative from the local Institutional Review Board at Indiana University, Washington University, Johns Hopkins University, the NIMH Intramural Research Program, University of Pennsylvania, University of California at Irvine, University of Iowa, University of Chicago, University of California at San Diego, and Rush University. After a detailed description of study participation, written informed consent was obtained for each subject.

### Subject Ascertainment

For genotyping as part of the BiGS, bipolar I subjects of European Ancestry were selected from those collected by the NIMH Genetics Initiative for Bipolar Disorder in five waves at 11 sites across the United States as described elsewhere in detail [8]. Recruitment for Waves 1 and 2 consisted of extended multiplex families ascertained through a bipolar I (BPI) or schizoaffective, bipolar type (SA-BP), proband, whereas Waves 3 and 4 consisted of families with a BPI proband and at least one other sibling with BPI or SA-BP. Wave 5 consisted solely of unrelated BPI singleton cases. All subjects were interviewed using the Diagnostic Interview for Genetic Studies (DIGS) [9]. Information was obtained from other family informants and medical records and reviewed along with the interview by a panel of experienced clinicians to obtain a final best-estimate diagnosis. The complete DIGS interview, which includes detailed information regarding manic and depressive episodes, is available for all subjects genotyped by the BiGS.

Control subjects were selected from those ascertained through a NIMH-supported contract mechanism between Dr. Pablo Gejman and Knowledge Networks, Inc [10]. All subjects donated a blood sample and were given a medical questionnaire. The selected controls were matched for gender and ethnicity (i.e., European Ancestry) with the bipolar cases, and all control subjects who endorsed a history of bipolar disorder, psychosis, or major depression were excluded from our study.

### Genotyping and Cleaning

The initial sample was genotyped at the Broad Institute as part of the GAIN using the Affymetrix 6.0 (1 M SNP) array. A total of 1,001 bipolar cases, 1,033 controls, and 724,067 SNPs were available for analysis following an extensive QC process to eliminate subjects with ≥5% missing data and SNPs with ≥5% missing data, minor allele frequencies<0.01, and Hardy-Weinberg Equilibrium p<10^−6^ [8]. The second sample was similarly genotyped at TGEN and underwent a comparable QC process that resulted in 1,190 bipolar cases, 401 controls, and 728,187 SNPs available for analysis [11]. An additional round of QC performed on the merged GAIN and TGEN samples resulted in 703,012 passing SNPs.

### Phenotypes

Phenotypes were derived from the Phenome Database, which compiles data across the DIGS 2, 3, and 4 to arrive at a common set of variables for each subject and allow for diagnostic consistency among the different versions of the DIGS used throughout Waves 1–5 of this sample [12]. As part of the DIGS interview, bipolar subjects were queried as to whether their most severe mania was irritable or elated. Table 1 describes the numbers of bipolar subjects in the GAIN, TGEN, and combined datasets with irritable or elated mania, as well as the total number of bipolar cases and controls, that were available for each analysis following quality control. Irritable mania comprised approximately 14% of subjects in the GAIN sample and approximately 12% of subjects in the TGEN sample. Subjects with missing severe mania mood data and were excluded from these analyses (41 in GAIN and 43 in TGEN). Each sample was approximately 60% female with very similar gender distributions observed in subjects with irritable vs. elated mania.

**Table 1:** Description of the subjects in the GAIN, TGEN, and combined datasets that were available for analysis.

Sample	# GAIN	# TGEN	# Combined
Irritable Mania	117	121	238
Elated Mania	843	1026	1869
All Bipolars	1001	1190	2191
Controls	1033	401	1434

### Statistical Analyses

To assess genetic factors contributing to irritable mania in the GAIN dataset, this subject group was compared to bipolar subjects with elated mania in a case-only primary analysis. In order to differentiate those genetic factors that are unique to the irritable mania subtype from those that may modify the expression of mania in bipolar disorder, the irritable mania group was also compared to controls in a secondary analysis. These results were contrasted with those comparing bipolar subjects with elated mania to controls, which proved to be very similar to the overall bipolar vs. control analysis. The irritable vs. elated mania case-only analysis was then repeated in the independent TGEN sample for replication and in the GAIN-TGEN combined sample as part of a joint analysis. The combined sample has 80% power to detect a locus with an allele frequency of 0.2. All association analyses were performed using logistic regression in PLINK [13]. Genomic inflation factors for all analyses in the GAIN, TGEN, and combined samples ranged from 1.00 to 1.01, so no correction for population stratification was deemed necessary. Label-switching permutations were performed to assess the empirical significance of all results, since spurious associations may result from a sampling bias through the selection of a small subset of individuals.

Heritability analyses of irritable mania were conducted in families from NIMH Waves 1–4 using SOLAR v.4.2.0, conditioning on the proband as a correction for ascertainment bias [14]. The complete DIGS interview is available for these subjects as part of data distribution 4.0. Subjects were characterized as having irritable or elated mania during their most severe episode, and only the 569 families of European Ancestry with a proband selected for participation in GAIN were assessed. There were 1,233 subjects with BPI or SA-BP diagnoses and mania mood data in these families, 14.3% of which had irritable mania. Sex, age at interview, site of interview, and wave of data collection were investigated as potential covariates with no significant effects identified in this sample.

## Results

Prior to conducting the association analyses, we sought to establish a basis for irritable mania as a valid subphenotype of bipolar disorder. In the 569 NIMH families from which a proband was genotyped as part of GAIN, a heritability estimate of 32.6±4.8% (p<0.001) was observed for irritable mania. While data from the most severe manic episode was used to characterize subjects for all analyses, we investigated the stability of irritable mania across episodes in the combined GAIN and TGEN samples. Although less than half of the subjects had mood data available for an additional manic episode, mood appeared to remain stable across episodes in this sample with a concordance rate >80% among those with available data.

**Figure 1:**
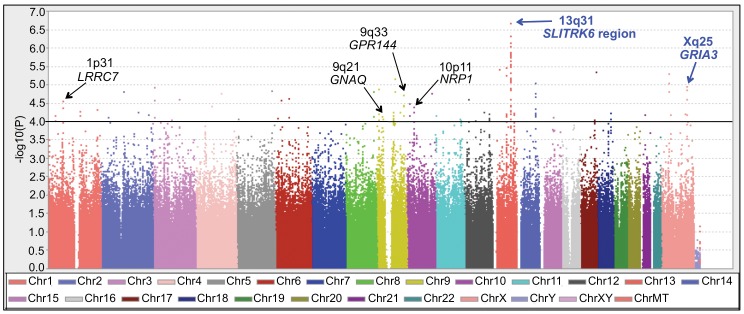
Genome-wide association results of the irritable vs. elated mania case-only analyses in the GAIN sample. The physical position is shown along the *x*-axis, color-coded by chromosome, and the –log (P value) for each SNP is shown along the *y* axis, as generated by Haploview 4.0. All regions containing at least two SNPs with p<10^−4^ and support for association from neighboring SNPs are indicated. Key: *LRRC7* = leucine rich repeat containing 7; *GNAQ* = guanine nucleotide binding protein; *GPR144* = G protein-coupled receptor 144; *NRP1* = neuropilin 1; *SLITRK6* = slit and trk like 6; *GRIA3* = AMPA3 ionotropic glutamate receptor.

The genome-wide results of the GAIN irritable vs. elated mania analysis are displayed in Figure 1. Genomic regions of interest were defined as those with at least two SNPs with p<10^−4^ and additional support for association (i.e., p<10^−3^) from surrounding SNPs within 100 kb. Several such regions were identified and confirmed through permutation analyses. The six regions meeting these criteria that were located within or near genes are also indicated in Figure 1. The SNPs within these regions with p values<10^−4^ are detailed in Table S1 with a comparison of the statistics from the irritable vs. elated, irritable vs. control, and elated vs. control analyses. All associations in the irritable vs. elated comparison were also prominent in the irritable vs. control comparison and not significant (p>0.05) in the elated vs. control comparison. The results of the bipolar vs. control analyses, as described elsewhere in detail [8], did not differ significantly from the elated vs. control and are not reiterated here.

**Figure 2:**
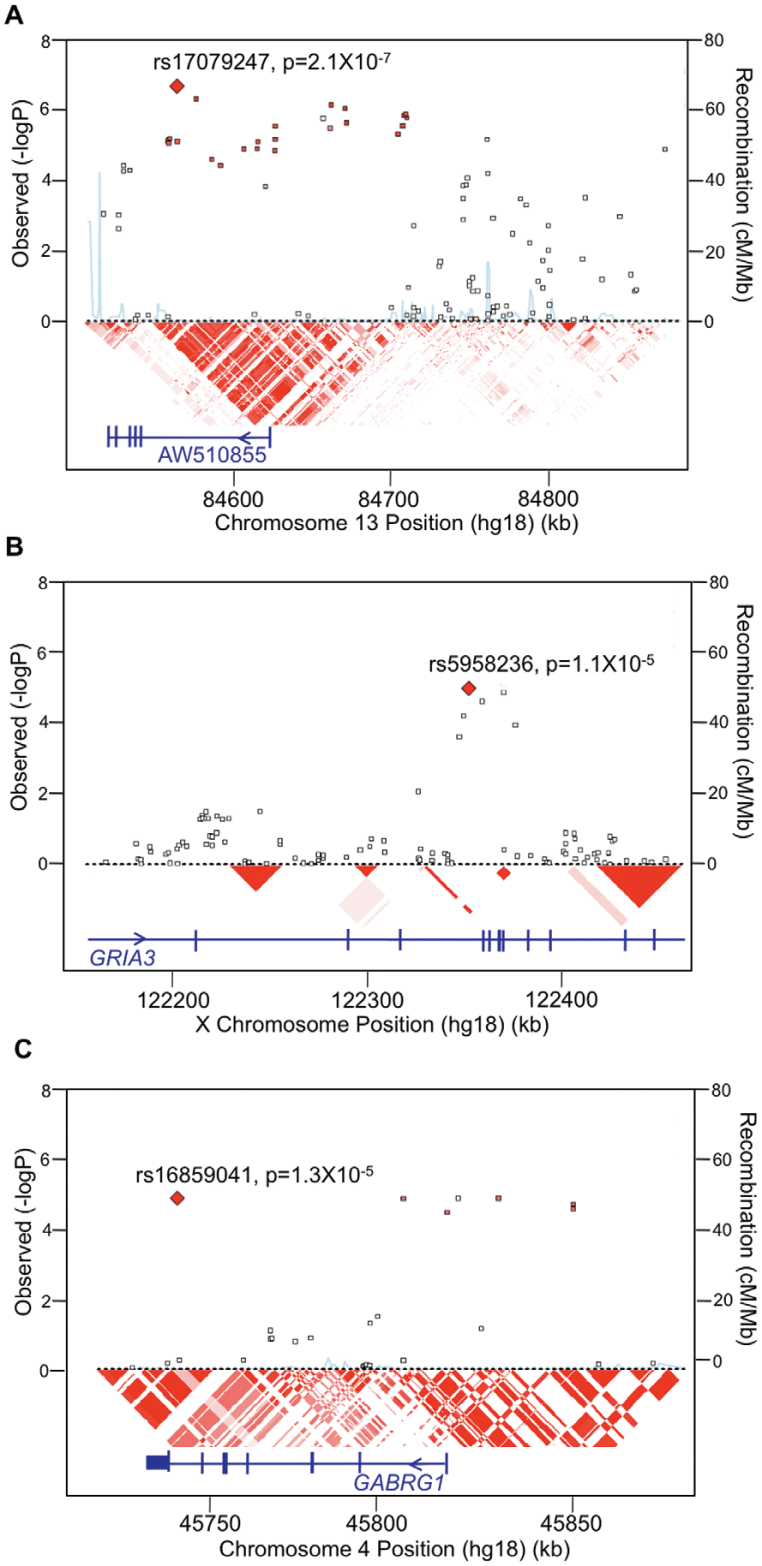
Details of the association results on chromosome 13 (A) and the X chromosome *GRIA3* gene region (B) in the GAIN irritable vs. elated mania case-only analysis, as generated by SNAP 2.0. The results for the chromosome 4 *GABRG1* gene region in the GAIN irritable mania vs. control analysis are shown in (C). Physical position and gene annotations according to HapMap release 22 are shown along the *x-axis*, and −log (P value) is shown on the left *y-axis*. The most significant SNP is indicated as a large red diamond, and all other SNPs are colored according to linkage disequilibrium levels (*r^2^*) with the primary SNP, as calculated from HapMap Release 22 CEU population data, with darker red representing higher values. Recombination rate within the CEU samples is shown on the right *y-axis* in blue. RefSeq genes are shown with all possible exons and arrows indicating transcript direction.

**Figure 3:**
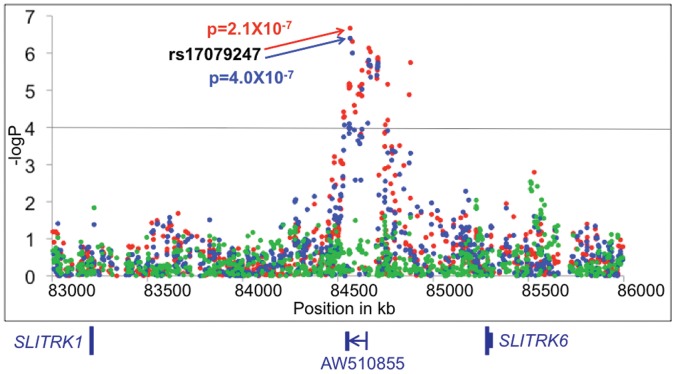
Results of the GAIN irritable vs. elated mania case-only analyses (red), the irritable mania vs. control analyses (blue), and the elated mania vs. control analyses (green) for the chromosome 13q31 region. The physical position is shown along the *x*-axis with the –log of the p value for each SNP is shown along the *y*-axis. The locations of the *SLITRK1* and *SLITRK6* genes are indicated below, along with a spliced EST.

The most significant genome-wide finding in the GAIN irritable vs. elated case-only analysis was for a region on chromosome 13q31 approximately 600 kb upstream of the *SLITRK6* gene (rs17079247, p = 2.1×10^−7^, odds ratio (OR) = 2.39). Support for this region came from a total of 33 SNPs spanning approximately 350 kb with p values<10^−4^, many of which exhibited high levels of LD with the most significant SNP, rs17079247 (see Figure 2A). These 33 SNPs were also prominent in the irritable vs. control analysis (peak p = 4×10^−7^ for rs17079247, OR = 2.31) and not significant (p>0.05) in the elated vs. control analysis (see Figure 3). The minor allele frequencies in the irritable mania group were generally double those of the elated mania or control groups. Comparison of the irritable group with all others in the sample (i.e., elated and control combined) resulted in even stronger support for this region with a peak p value 7.3×10^−8^, nearing conventional genome-wide significance. Although there are no known genes directly beneath this peak, rs17079247 is located within a spliced EST (AW510855) and a *TAF1* binding site, and this region has been suggested to contain a novel gene not yet described, as well as a CpG island [15], [16]. Since the peak is located between the *SLITRK1* and *SLITRK6* genes with *SLITRK5* further downstream and no other genes nearby, it is also possible that this region contains a regulatory element for this gene cluster from the *SLITRK* family, all of which are expressed in neural tissues and function to suppress neurite outgrowth [17].

The second most interesting finding in the case-only analysis was within the *GRIA3* gene on chromosome Xq25 with a peak p value of 1×10^−5^ (OR = 1.99). P values<10^−4^ were observed for three other SNPs in this region, which did not reveal appreciable levels of LD with the most significant SNP, rs5958236 (see Figure 2B). These four SNPs within the *GRIA3* gene show the same OR pattern, were generally prominent in the irritable vs. control analysis (peak p = 5.8×10^−4^), and were not significant in the elated vs. control analysis. Since *GRIA3* encodes a glutamate receptor subunit that forms a ligand-gated ion channel responsive to glutamate, the predominant excitatory neurotransmitter in the brain, this gene may be of relevance to bipolar disorder and irritable mania in particular.

Several genomic regions of interest were also identified in the GAIN irritable vs. control analysis, most of which overlapped with the irritable vs. elated mania case-only analysis (see Table S1). This analysis did, however, identify two additional regions of interest, one of which was the *CNTN6* gene on chromosome 3p26 (peak p = 5.5×10^−6^), which belongs to a family of cell adhesion molecules involved in the formation of axon connections during nervous system development [18]. The *GABRG1* gene on chromosome 4p12 was also implicated with a total of four SNPs within or just upstream of the gene revealing p values of 1.3×10^−5^ (OR = 3.45) and four additional SNPs in the region exhibiting p values<10^−4^ with similar ORs (see Figure 2C). Support for association to *GABRG1* was also observed in the irritable vs. elated case-only analysis with a peak p value of 1.4×10^−4^. *GABRG1* opens a chloride channel in response to binding GABA, the major inhibitory neurotransmitter in the brain. Interestingly, many anticonvulsants, which are an effective treatment for irritable mania, target the GABA system.

We attempted to replicate these results with an analysis of irritable vs. elated mania in the independent TGEN sample. While several genomic regions met our set criteria for consideration (see Figure S1), none of these regions overlapped with the GAIN results. In particular, the region on 13q31 most associated in the GAIN sample was not significant (p>0.05) in the TGEN sample, although the effect was in the same direction (OR = 1.3). A further examination of the GAIN and TGEN samples revealed significant differences in the prevalence of co-morbid disorders, with the TGEN sample exhibiting significantly higher levels of co-morbid phobias, obsessive-compulsive disorder (OCD), eating disorders, rapid cycling, and psychosis (see Table 2). These differences seemed to stem primarily from differences between bipolar subjects collected as part of a family-based sample (Waves 1–4), which comprised two-thirds of the GAIN sample, as opposed to those collected as singleton cases (Wave 5), which comprised the remainder of the GAIN sample and the entire TGEN sample. For this reason, the GAIN sample may include subjects with greater genetic loading and hence greater power to detect associated loci. We note that significant differences in co-morbidity were not observed between bipolar subjects with irritable vs. elated mania in either sample, nor were differences observed for gender, age at interview, age at onset, age at first mania, or age at most severe mania for any comparison (i.e., irritable vs. elated mania, GAIN vs. TGEN, family vs. singleton, all p>0.10). These differences in clinical profile suggest that the different methods of ascertainment may have led to a sampling of two genetically, and possibly mechanistically, different groups of bipolar patients.

**Table 2:** Summary of the observed frequencies differences of co-morbid disorders between the GAIN and TGEN samples, as well as between the underlying family and bipolar singleton samples.

	GAIN	TGEN	GAIN vs. TGEN	Family	Singleton	Family vs. Singleton
Co-morbid Disorder	Freq	Freq	P Value	Freq	Freq	P Value
Alcoholism	46.3	49.0	0.218	**42.4**	**49.6**	**0.004**
**Substance Abuse**	**14.7**	**18.1**	**0.033**	**11.3**	**18.4**	**<0.001**
Panic Disorder	23.9	27.0	0.105	**22.1**	**26.8**	**0.031**
**Agoraphobia**	**12.2**	**17.3**	**0.001**	**9.8**	**16.8**	**<0.001**
**Simple Phobia**	**9.6**	**15.3**	**<0.001**	**7.6**	**14.5**	**<0.001**
**Social Phobia**	**10.0**	**15.1**	**<0.001**	**7.4**	**14.6**	**<0.001**
**Obsessive Compulsive Disorder**	**7.8**	**13.5**	**<0.001**	**6.6**	**12.6**	**<0.001**
**Anorexia/Bulimia**	**6.1**	**10.5**	**<0.001**	**5.4**	**9.6**	**0.002**
**Rapid Cycling**	**57.4**	**63.3**	**0.006**	**50.9**	**63.9**	**<0.001**
**Passive Death Wish**	**72.9**	**78.2**	**0.007**	74.1	76.3	0.314
**Psychosis**	**66.6**	**71.8**	**0.010**	**61.8**	**72.1**	**<0.001**

Key: Family refers to bipolar subjects ascertained through the collection of families for NIMH Waves 1–4. Singleton refers to bipolar subjects ascertained as singleton cases collected as part of Wave 5. Note: Significant differences in co-morbidity were not observed between bipolar subjects with irritable vs. elated mania in either sample, nor were differences observed for gender, age at interview, age at onset, age at first mania, or age at most severe mania for any comparison (i.e., irritable vs. elated mania, GAIN vs. TGEN, family vs. singleton, all p>0.10).

In a joint analysis of the GAIN and TGEN samples for irritable vs. elated mania, rs17079247 in the *SLITRK6* gene region on chromosome 13q31 produced the peak p value of 7.7×10^−6^ (OR = 1.75) with 24 other SNPs in the region associated with p<10^−3^, despite the complete lack of association to this region in the TGEN sample. Other regions of interest identified in the GAIN sample were either not significant or only of nominal significance (p<0.05), as one would expect from the joint analysis of two samples exhibiting such marked clinical heterogeneity. The evidence for the chromosome 13q31 region in the GAIN analyses stemmed primarily from the 73 (of 117) subjects with irritable mania that were derived from the family-based sample (Waves 1–4) with a peak p value of 2.9×10^−6^ for rs17079247. Still, the association to this region remained with a p value<10^−5^ in the joint analysis, despite the clinical heterogeneity between the samples. This may suggest that least some portion of the singleton cases in TGEN, perhaps those with lower co-morbidity rates comparable to the family-based samples, provided support for association to this region.

## Discussion

Since bipolar patients display significant clinical variability, which may reflect underlying genetic heterogeneity, the use of DSM diagnosis of bipolar disorder as a phenotype may not have the best power to detect causal genes. The use of selected clinical features as subphenotypes to create more homogenous subgroups of patients may aid in the identification of genes. We have investigated the possible utility of irritable mania as a subphenotype of bipolar disorder, finding evidence for significant heritability, inter-episode stability, and an apparently distinct genetic profile.

The most significant finding in the primary GAIN analysis of irritable vs. elated mania was for a region on chromosome 13q31 between the *SLITRK1* and *SLITRK6* genes with a peak p value of 2.1×10^−7^ and a total of 33 SNPs providing support with p values<10^−4^. A p value of 7.3×10^−8^ was further observed when the irritable group was compared with all other subjects in the sample (elated and control combined). This region also featured prominently the analysis of irritable mania vs. controls with a peak p value of 4.0×10^−7^. We note that the inclusion of a small percentage of subjects (<5%) with SA-BP did not impact these analyses, since the prevalence of irritable mania in SA-BP subjects was similar to that for BPI subjects, and the removal of these subjects produced comparable results. While the clinically diverse TGEN sample failed to replicate this finding, the 13q31 region was still the most prominent finding in the joint analysis with a peak p value of 7.7×10^−6^, despite the high degree of clinical heterogeneity between the two samples. This region is of particular interest in light of linkage analyses that have implicated chromosome 13q31 in bipolar families segregating psychotic symptoms [19], as well as linkage to the nearby 13q32 region for bipolar disorder [20]–[22]. Associations to *SLITRK1* have also been reported for Tourette’s Syndrome [23] and cyclothymic temperament in bipolar disorder [24]. Intriguingly, the region directly below the association peaks has been suggested to contain a novel gene not yet described, possibly evidenced by the presence of a spliced EST [15]. However, with the nearest known gene, *SLITRK6*, located approximately 600 kb away, it is conceivable that this region could potentially harbor a regulatory element that mediates the expression of the *SLITRK* gene family cluster of *SLITRK1*, *SLITRK5*, and *SLITRK6*, all of which function in neurodevelopment to suppress neurite outgrowth [17]. Additionally, it is possible that the observed association may be related to structural variation in the region, as has been reported for this sample [25].

Previous GWA studies of bipolar disorder have implicated several potential susceptibility genes, such as *SYNE1* on chromosome 6q25.2, *ODZ4* on 11q14.1, *ANK3* on 10q21.2, *CACNA1C* on 12p13.3, and *NCAN* on 19p13.1 [26]–[28]. While none of these genes featured prominently in any of the analyses presented here, modest support for a few of the genes was observed. Only nominally significant p values (<0.05) for SNPs within *SYNE1* were observed in both GAIN analyses involving irritable mania. The TGEN analysis, however, identified two SNPs within the gene with p values<10^−4^, and the GAIN elated mania analysis identified association to several SNPs with p values<0.001. Similarly, for *ANK3*, only nominally significant p values (<0.05) for SNPs within the gene were observed in any of the analyses involving irritable mania, although the GAIN elated mania analysis identified several SNPs with p values<0.001. These results are not surprising, given that the analysis of elated mania vs. controls approximated that of bipolar disorder as a whole. No evidence of association (p>0.05) with any SNP within or near *CACNA1C*, *ODZ4*, or *NCAN* was found in any of our analyses.

There has been much debate over the appropriate threshold for reporting genes and associations from a GWA study that are likely to be genuine findings of interest. For example, the landmark GWA study of the Wellcome Trust Case Control Consortium in 2007 used a Bayesian method to establish 5×10^−7^ as a level of “strong evidence of association” in a GWA study and indicated that SNPs with nominal p values of 10^−4^–10^−7^ were considered of interest [29]. In our analyses of the GAIN sample, the region of association on chromosome 13q31 surpasses this level in both the irritable vs. elated mania and irritable mania vs. control analyses, with peak p values of 2.1×10^−7^ and 4.0×10^−7^, respectively. We also identified several genomic regions associated with irritable mania with multiple p values in the range of 10^−4^–10^−7^. These regions were not significant in the analyses of elated mania vs. controls or of bipolar disorder vs. controls [8], suggesting that they may harbor susceptibility genes particular to the irritable mania subtype of bipolar disorder. Due to the small sample size of our irritable cohort, we lacked sufficient power in the primary GAIN analyses to detect a p value meeting the conventional genome-wide significance criteria of 5×10^−8^, and the estimation of p values may be distorted in small samples. However, permutation analyses performed to assess the empirical significance of the results provided similar estimates of association (see Table S1), lending confidence to our results, despite the limitations introduced by the sample size.

These data provide support for the concept that different clinical manifestations of bipolar disorder may reflect differences in the underlying genetic architecture. In particular, we have found evidence to suggest a distinct genetic profile for the irritable presentation of mania. A comprehensive evaluation of the utility of mania subphenotypes, and of irritable mania in particular, in genetic studies of bipolar disorder will require further study in additional and larger samples.

## Supporting Information

Figure S1 Genome-wide association results of the irritable vs. elated mania case-only analyses in the TGEN sample. The physical position is shown along the *x*-axis, color-coded by chromosome, and the –log (p value) for each SNP is shown along the *y*-axis, as generated by Haploview 4.0. All regions containing at least two SNPs with p<10^−4^ and support for association from neighboring SNPs are indicated. Key: *RABGAP1L = *RAB GTPase activating protein 1-like*; RPS6KC1 = *ribosomal protein S6 kinase, 52 kDa, polypeptide*; GALNT2 = *polypeptide N-acetylgalactosaminyltransferase 2*; CNTN3 = *contactin 3; *KCND2 = *potassium voltage-gated channel, Shal-related subfamily, member 2*; MSRA = *methionine sulfoxide reductase A*; RIMBP2 = *RIM-binding protein 2*; CLCN4 = *chloride channel 4.
(TIF)

Table S1 Summary of the most significant SNPs in the GAIN analysis of irritable vs. elated mania with comparison to the results of the irritable mania vs. control and elated mania vs. control analyses. Key: Chr = chromosome; A1/A2 = alleles 1 (major) and 2 (minor); MAF = minor allele frequency; IM = irritable mania; EM = elated mania; CTL = control; OR = odds ratio; P = p value; Perm P = permuted p value.
(XLS)
