# Supplementary figures and images for: Genome-Wide Association Study of Irritable vs. Elated Mania Suggests Genetic Differences between Clinical Subtypes of Bipolar Disorder
Source: PLoS One. 2013 Jan 10;8(1):e53804. doi: 10.1371/journal.pone.0053804 (PMC3542199; doi:10.1371/journal.pone.0053804)

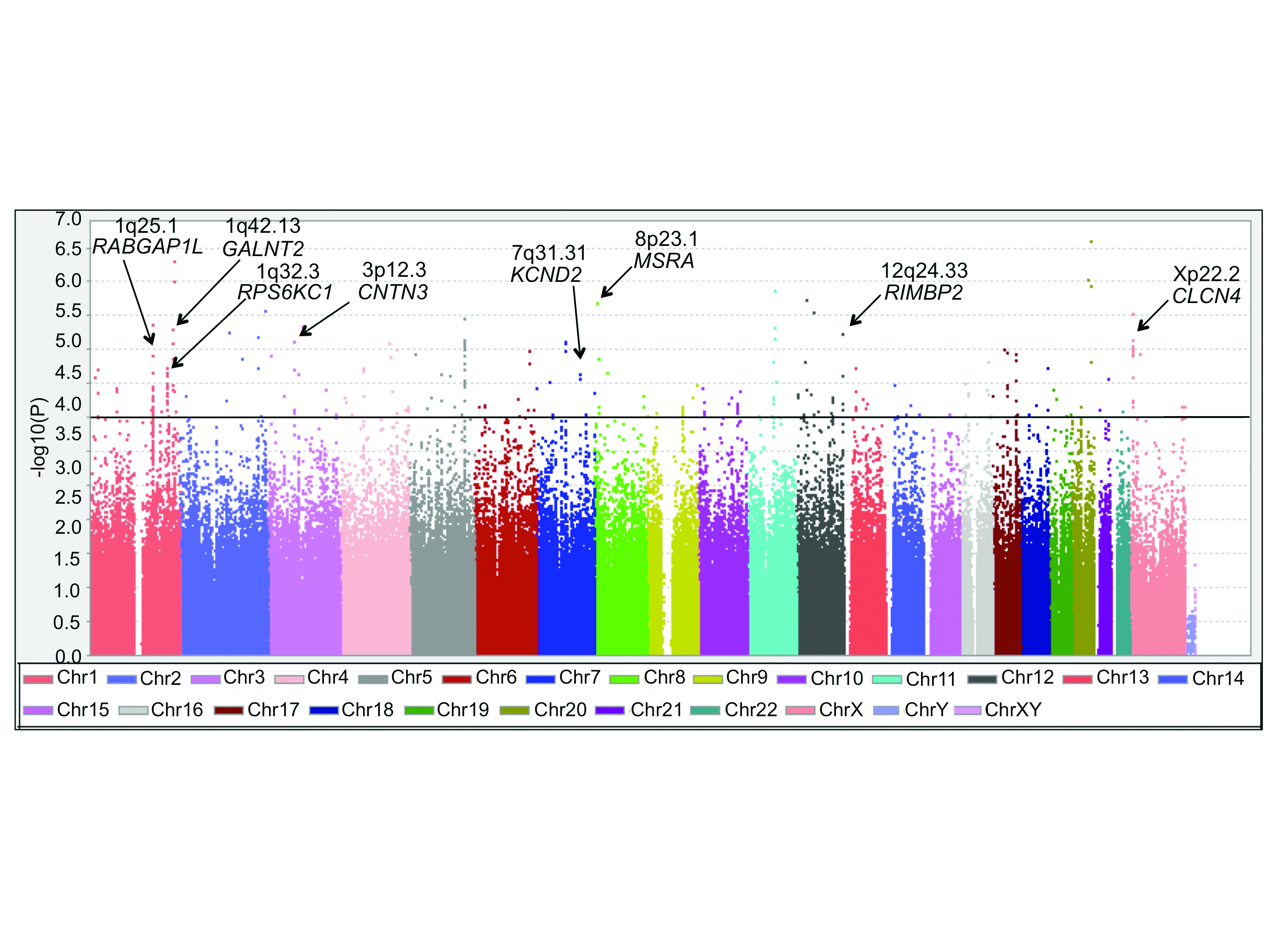

Supplement: Figure S1 — Genome-wide association results of the irritable vs. elated mania case-only analyses in the TGEN sample. The physical position is shown along the x-axis, color-coded by chromosome, and the –log (p value) for each SNP is shown along the y-axis, as generated by Haploview 4.0. All regions containing at least two SNPs with p<10−4 and support for association from neighboring SNPs are indicated. Key: RABGAP1L = RAB GTPase activating protein 1-like; RPS6KC1 = ribosomal protein S6 kinase, 52 kDa, polypeptide; GALNT2 = polypeptide N-acetylgalactosaminyltransferase 2; CNTN3 = contactin 3; KCND2 = potassium voltage-gated channel, Shal-related subfamily, member 2; MSRA = methionine sulfoxide reductase A; RIMBP2 = RIM-binding protein 2; CLCN4 = chloride channel 4. (TIF) [file pone.0053804.s001.tif]
